# Supplementary material for: Apicortin defines the Plasmodium apical conoid body but is dispensable for the parasite life cycle
Source: Life Sci Alliance. 2026 Jan 16;9(4):e202503522. doi: 10.26508/lsa.202503522 (PMC12811414; doi:10.26508/lsa.202503522)
Supplement: Supplementary file 1 [file LSA-2025-03522_SdataFS1.1_FS2.1.docx]

**S1_Raw_images supporting gels**

DNA gel used in Fig S1B. Lanes 1 (wildtype control) and 2 (tagged cell line) show PCR results for gene-specific GFP integration, lanes 3 (wildtype control) and 4 (tagged cell line) show positive control PCR. M is marker lane. All unboxed lanes are unrelated to this report.


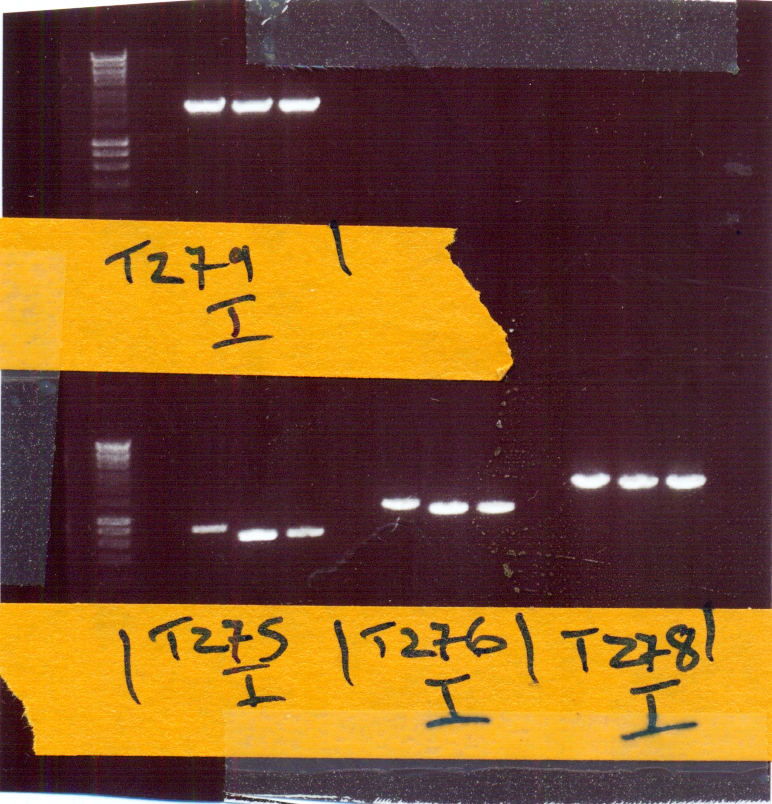


M

3

4

2

1

DNA gel used in Fig S2B. Lanes 1 (wildtype control) and 2 (KO cell line) show PCR results for gene-specific integration; lanes 3 (wildtype control) and 4 (KO cell line) show gene deletion PCR; lanes 5 (wildtype control) and 6 (KO cell line) show positive control PCR. M is marker lane. All unboxed lanes are unrelated to this report.


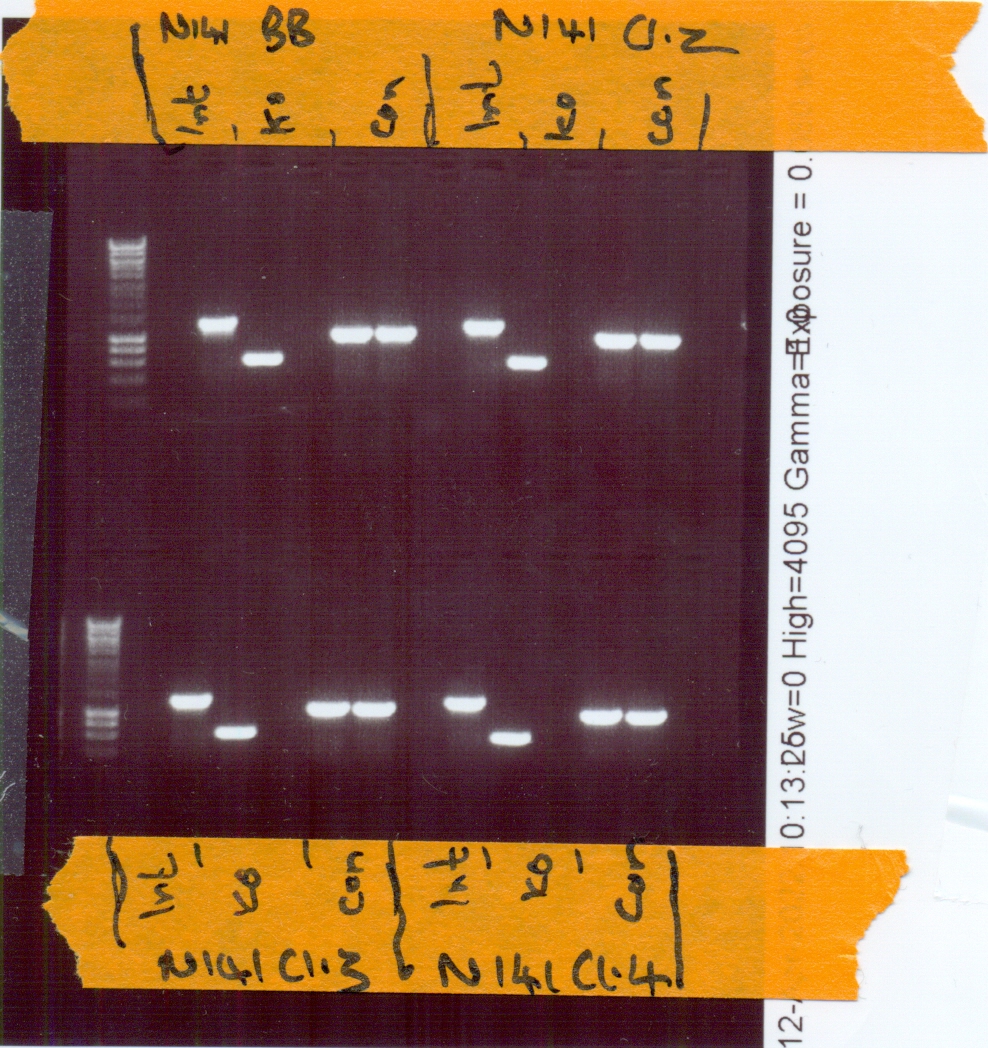


5

6

3

4

2

M

1
